# Clinical Findings and Outcome in 30 Dogs with Presumptive or Confirmed Nerve Sheath Tumors

**DOI:** 10.3390/vetsci11050192

**Published:** 2024-04-28

**Authors:** Rachel S. Cooper-Khan, Alexandra N. Frankovich, Craig A. Thompson, Stephanie A. Thomovsky, Melissa J. Lewis

**Affiliations:** 1 Department of Veterinary Clinical Sciences, Purdue University College of Veterinary Medicine, 625 Harrison Street, West Lafayette, IN 47907, USA; coope308@purdue.edu (R.S.C.-K.); sthomovs@purdue.edu (S.A.T.); 2 Department of Comparative Pathobiology, Purdue University College of Veterinary Medicine, 625 Harrison Street, West Lafayette, IN 47907, USA; francovi@purdue.edu (A.N.F.); cathomps@purdue.edu (C.A.T.); 3 Department of Clinical Sciences, College of Veterinary Medicine, North Carolina State University, 1060 William Moore Drive, Raleigh, NC 27606, USA

**Keywords:** malignant nerve sheath tumor, trigeminal nerve, brachial plexus, magnetic resonance imaging, fine needle aspiration, canine

## Abstract

**Simple Summary:**

Nerve sheath tumors (NSTs) are well-recognized tumors of the nervous system, but there is relatively limited information about these tumors in dogs. This study describes a group of dogs with NSTs affecting the nerves associated with the head or the spinal cord. Thirty dogs were included, 25 with a presumptive diagnosis and five with a confirmed diagnosis. Eight dogs had NSTs affecting one or more nerves of the head including five where the tumor caused compression of the brain. Six involved the trigeminal nerve. Twenty-two dogs had tumors affecting one or more spinal nerves with the majority affecting the nerves of a front limb. In 13 of the 22 dogs, the tumor caused compression of the spinal cord. The outcome was poor overall. Among the dogs alive at least 1 week after diagnosis, the survival time ranged from weeks to years. All dogs were eventually euthanized because of disease progression. While there was a broad overlap between tumor locations, survival was generally longer for dogs without compression of the spinal cord or brain. The results are limited by the small number of dogs with a confirmed diagnosis but still expand what is known about NSTs in dogs and highlight the need for more information on predictors of outcome.

**Abstract:**

Nerve sheath tumors (NSTs) are well-recognized primary nervous system tumors, but there is relatively limited information in dogs including comparison of NSTs in different anatomical locations. This retrospective study describes the clinical features and outcomes in a group of dogs with NSTs affecting the cranial nerves or spinal nerves. Thirty dogs were included, 25 with a presumptive diagnosis and five confirmed by histopathologic analysis. Seven dogs also had cytology of tumor samples, which were supportive of the NST diagnosis in four. Eight dogs had cranial nerve-associated NSTs, with six involving the trigeminal nerve. Twenty-two dogs had spinal nerve-associated NSTs including 13 invading the spinal canal and nine peripheral to the spinal canal, with the majority affecting nerves or nerve roots of the brachial plexus. The prognosis was poor, with dogs being euthanized eventually because of disease progression. Among dogs alive 1 week after diagnosis, the median survival time was 4 months but ranged from 2 weeks to >2 years. While there was a broad overlap between NST locations, survival was generally longer for dogs without spinal canal or intracranial involvement. The results expand available information on NSTs in dogs but should be interpreted with caution given the small number of dogs with a definitive diagnosis. Further investigation is warranted to determine how tumor location, invasiveness, and treatments pursued impact outcome.

## 1. Introduction

Nerve sheath tumors (NSTs) are neoplasms that arise from either Schwann cells or the associated connective tissue surrounding the nerve fascicles or individual axons of the peripheral nervous system, including cranial nerves (CNs) [1]. The cause of NSTs in dogs is unknown. A point mutation of the neu oncogene might be responsible for some malignant NSTs in several animal species including dogs [2], but the etiology has not been identified for most NSTs. Additionally, it can be difficult to determine the specific cell of origin in dogs. While an updated histologic and immunohistochemical classification has been recently proposed for dogs, the broader terms NST or malignant peripheral nerve sheath tumor are typically used [3].

NSTs can occur throughout the body, and they can involve both cranial nerves and nerves associated with the spinal cord [4,5,6,7,8,9,10,11,12,13]. In dogs, NSTs commonly involve the nerve roots or nerves of the brachial plexus in the caudal cervical and cranial thoracic region [5,7,9,14,15,16] and the trigeminal nerve [6,17,18]. They tend to have a low rate of distant metastasis but are extensively locally aggressive and invasive [1]. This can result in multiple nerves or nerve roots being affected at once. In addition to peripheral nerve dysfunction, clinical signs of a myelopathy or intracranial dysfunction are possible with spinal cord or brain invasion, respectively [5,9,16,17]. Definitive diagnosis is based on histopathologic analysis of the tumor [3] though presumptive diagnosis can be achieved using characteristic clinical presentation and imaging findings [17,18,19,20,21,22,23,24]. Treatment options depend on location, and prognosis is variable but is generally considered to be poor because of their locally aggressive behavior [5,6,9,14,17,25,26,27,28,29].

Despite being well-recognized primary tumors of the nervous system in dogs, there is limited information available regarding the clinical features and course of NSTs in different locations. This retrospective study aimed to describe the clinical findings and outcomes in a group of dogs with NSTs affecting the spinal nerves or cranial nerves.

## 2. Material and Methods

For this retrospective study, the medical record database of the Purdue University Veterinary Hospital was searched from 2012 to 2021 for dogs diagnosed with an NST. Cases were identified using the following search terms: peripheral nerve sheath tumor, PNST, nerve sheath tumor, NST, malignant nerve sheath tumor, MNST, schwannoma, neurofibroma, neurofibromatosis, nerve root tumor, brachial plexus tumor, lumbar plexus tumor, cervical intumescence tumor, lumbar intumescence tumor, cervical nerve root tumor, lumbar nerve root tumor, and sacral nerve root tumor. To be included, dogs had to have a presumptive (based on MRI) or confirmed (based on MRI and histopathology) diagnosis of an NST. Diagnosis of NST using MRI was made based on previously published criteria including diffuse nerve thickening or a discrete mass in the region of a nerve that was hyperintense on T2W imaging, isointense on T1W imaging, and variably contrast enhancing [1,17,19,20,24]. Histopathology was performed on surgical biopsies or necropsy tissue samples from the mass, and a diagnosis was made at the discretion of board-certified pathologists using previously published criteria [3,30]. The results of special stains were reported when performed but were not a requirement to be classified as having a confirmed diagnosis. Cases were excluded if the diagnosis was something other than a presumptive or confirmed NST or if the medical record contained insufficient information to make this determination.

The following information was collected from the medical record: age, weight, sex, breed, onset and duration of clinical signs prior to presentation, neurologic examination findings at diagnosis, mass location (based on MRI or necropsy), imaging findings, treatments administered, and outcome. When performed, electromyography (EMG) results, cerebrospinal fluid (CSF) analysis, and histopathologic and/or cytologic analysis were also recorded. Duration was defined as the time elapsed between the onset of clinical signs and diagnosis. Mass location within the nervous system was described as cranial (i.e., cranial nerve associated with or without intracranial extension), peripheral spinal (i.e., mass associated with one or more nerves or nerve roots located external to the spinal canal), or invasive spinal (i.e., mass associated with one or more nerves or nerve roots with extension into the spinal canal). Masses that extended into one or more intervertebral foramina but lacked overtly visible extension into the spinal canal on MRI were grouped with the peripheral spinal group.

Cytology was performed on tumor fine needle aspirates (FNAs) or squash preparation of tumor biopsy samples. Cytologic findings were described as consistent with neoplasia, consistent with a sarcoma specifically or non-diagnostic. Treatments were categorized as palliative, definitive, or none. Palliative treatment was defined as analgesic medications and/or corticosteroid administration, and definitive treatment included radiation therapy and/or surgery of any kind. Survival time was defined as the time from diagnosis to death. No survival time was recorded if the patient was lost to follow-up. The date and cause of death or euthanasia were recorded when available. Primary veterinarians and owners were contacted to gather additional follow-up information when indicated.

All data are presented descriptively with mean (and standard deviation) or median (and range), as appropriate.

## 3. Results

**Figure 1:**
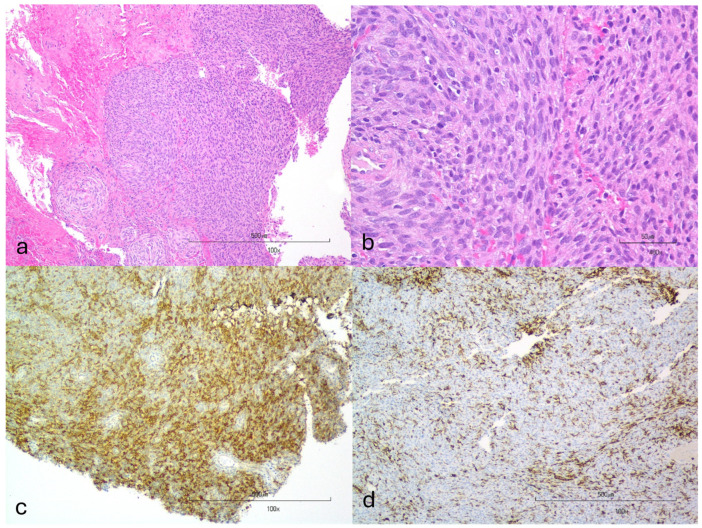
Photomicrographs from an intra-operative biopsy sample from a dog with a confirmed invasive spinal NST at the level of C2–C3. (**a**) In the center of the image is a bundle of neoplastic cells surrounded by dura mater and loose fibrovascular stroma. H&E stain. (**b**) Bundles and streams of ovoid to fusiform neoplastic cells in scanty fibrous stroma are present and, in some fields, neoplastic cells form spherical aggregates of rounded to polyhedral cells. H&E stain. (**c**) Neoplastic cells have strong cytoplasmic CNPase immunoreactivity. CNPase IHC. (**d**) Many macrophages throughout the stroma have strong cytoplasmic IBA-1 immunoreactivity, but neoplastic cells do not have IBA-1 immunoreactivity. IBA-1 IHC. The cellular architecture and immunohistochemistry staining results are most supportive of a nerve sheath tumor.

**Figure 2:**
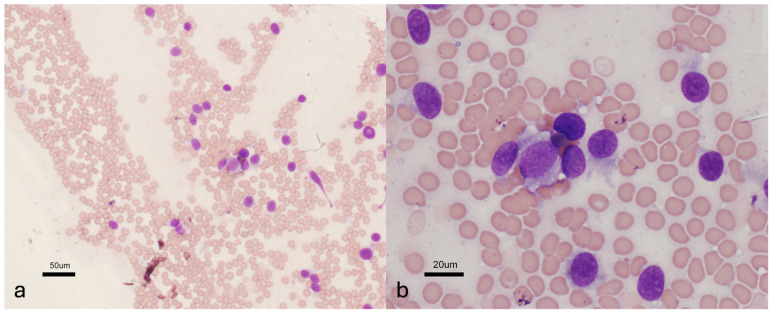
Cytologic images from a dog with a confirmed invasive spinal NST at the level of C2–C3. Images at 20× (**a**) and 60× (**b**) magnification show neoplastic cells scattered about in a light background of blood. The cells are individualized and have a single, or occasionally two, round- to ovoid-shaped nuclei set in scant to a small amount of blue cytoplasm that often has ill-defined, wispy margins. The nuclei exhibit a grainy chromatin pattern. Findings are consistent with a spindle cell neoplasm. Modified Wright-Giemsa stain.

Thirty dogs were included with a presumptive (N = 25) or confirmed (N = 5) diagnosis of an NST. Eight (27%) dogs had cranial NSTs and twenty-two (73%) had spinal NSTs, 13 invasive and nine peripheral. Of the five dogs with confirmed NSTs, four were diagnosed via histopathology alone (two cranial NSTs via necropsy and two peripheral spinal NSTs via surgical biopsy). One invasive spinal NST was diagnosed via histopathology from surgical biopsy and necropsy samples with additional immunohistochemistry performed (CNPase-positive, CD18-negative) to rule out other tumor types (Figure 1). Seven dogs had cytology performed on tumor samples. Four of seven dogs had cytologic results that supported the NST diagnosis including malignant neoplasia in one (invasive spinal NST) and sarcoma in three (one invasive spinal and two peripheral spinal) (Figure 2). Cytologic diagnosis of sarcoma was confirmed histologically and immunohistochemically to be an NST in one dog with an invasive spinal NST. Three of seven ultrasound-guided FNA samples were non-diagnostic (two peripheral spinal and one cranial NST).

The mean age for all dogs was 8.6 ± 2.6 years, and the median weight was 20.25 kg (4.1 to 45.4 kg). There were 13 spayed females, one intact female, 14 neutered males, and two intact males. There were 18 breeds represented; the most common breeds were Labrador retriever (n = 5) and mixed-breed dog (n = 8). The other 16 breeds were represented by two or fewer cases. The duration of clinical signs ranged from 1 week to 2 years, with a median of 2 months.

Twenty-eight out of thirty (93%) dogs received some form of treatment for their NST, with the two untreated dogs euthanized at 0 and 2 days after diagnosis. All 28 dogs were administered palliative treatment, and 6 dogs received additional definitive treatment consisting of radiation therapy (n = 4), surgery (n = 1), or a combination of both therapies (n = 2). Three cases were lost to follow-up at 0 days, 2 months, and 1.5 years after diagnosis while the remaining 27 dogs were humanely euthanized. Seven dogs were euthanized within a week of diagnosis. Among dogs alive at least a week from diagnosis, the median survival time from diagnosis to euthanasia was 4 months, ranging from 2 weeks to 2 years and 8 months.

### 3.1. Cranial NSTs

Eight of thirty (27%) dogs were diagnosed with cranial NSTs, two being confirmed and six presumptive (Table 1). The trigeminal nerve was affected in six dogs (three right and three left), whereas multiple cranial nerves (glossopharyngeal, vagus, and accessory nerves) were affected in two dogs. In five dogs, MRI revealed intracranial involvement, whereas only the peripheral components of the trigeminal nerve were overtly involved in three dogs. The mean age was 8.5 ± 3.0 years, and the weight ranged from 10.2 to 36.0 kg, with a median of 20.3 kg. There were five spayed females and three neutered males. Breeds included mixed breed (n = 4), Labrador retriever (n = 2), French bulldog (n = 1), and beagle (n = 1). The duration of clinical signs ranged from several weeks to 1 year. The most common presenting complaints were unilateral facial muscle atrophy (n = 4), head tilt (n = 4, including a transient, intermittent head tilt in 1), and ocular signs (n = 3). One dog was presented for seizure activity. Of the dogs presenting with head tilts, two had otitis media/interna ipsilateral to a mass affecting the trigeminal nerve and two had no visible bulla abnormalities, one with a mass affecting the trigeminal nerve, and the other with a mass affecting glossopharyngeal, vagus, and accessory nerves.

Neurological abnormalities at diagnosis consisted of one or more cranial nerve deficits in all dogs including unilateral masticatory muscle atrophy (n = 6), unilateral decreased to absent facial sensation (n = 4), unilateral decreased to absent menace response (n = 4), miosis in one or both eyes (n = 3), head tilt (n = 3), and decreased to absent gag (n = 1). Four dogs had intracranial signs, all of which had intracranial extension evident on MRI, while one additional dog with clinical signs of an isolated trigeminal neuropathy had intracranial involvement on MRI. EMG was not performed in any dogs with cranial NSTs. Two dogs had CSF analysis performed, which revealed protein cytologic dissociation in one (total protein = 49 mg/dL, total nucleated cell count = 5 cells/µL) and a pleocytosis in the other (total protein = 35 mg/dL, total nucleated cell count = 24 cells/µL, 50% large mononuclear cells and 50% small mature lymphocytes). No neoplastic cells were observed in CSF from either dog.

Seven of the eight dogs received some form of treatment, while one dog with intracranial extension evident on MRI was euthanized 2 days after diagnosis without treatment. Five dogs received palliative treatment alone with a median survival time from diagnosis to euthanasia of 4 months (range 3 weeks to 1 year and 11 months). Three palliatively treated dogs had intracranial extension on MRI with survival times ranging from 3 weeks to 4 months, while two palliatively treated dogs without intracranial extension on MRI had survival times of 5 months and 1 year and 11 months. Two dogs received a combination of palliative treatment and radiation therapy. One dog who underwent radiation therapy for a trigeminal NST with intracranial extension on MRI was reported by the owner to be doing well 1.5 years after diagnosis but was then lost to follow-up. The other dog with a trigeminal NST without intracranial extension on MRI underwent three separate rounds of stereotactic radiation along with palliative treatment and survived 2 years and 8 months.

### 3.2. NSTs with Spinal Canal Invasion

Thirteen of the thirty (43%) dogs were diagnosed with NSTs with spinal canal invasion, two being confirmed and 11 presumptive (Table 2). Affected nerve roots ranged from C1 to T3, with seven between C1 and C5 and six between C6 and T3. Based on MRI, 10 masses were classified as having extradural and intradural components, and 6 of these appeared to have additional intramedullary invasion. Two tumors were classified as intradural–extramedullary, and one tumor was classified as extradural but with components located within the spinal canal.

The mean age was 8.8 ± 2.4 years, and the weight ranged from 4.1 to 45.5 kg, with a median of 8.7 kg. There were five spayed females, one intact female, six neutered males, and one intact male. There were 13 different breeds, each represented by one dog. The duration of clinical signs ranged from 5 days to 1 year. The most common owner-reported clinical sign was progressive lameness (n = 10). Neurological abnormalities at presentation included pain (n = 9), paresis (n = 8), decreased reflexes of the affected limb (n = 12), and proprioceptive ataxia (n = 5). Eleven dogs had signs suggestive of myelopathy, while two dogs demonstrated only pain and progressive lameness.

EMG was not performed in any dogs with invasive spinal NSTs. CSF analysis on samples obtained from the cisterna magna was performed in 12 dogs. Two dogs had a pleocytosis (total protein = 71 mg/dL, total nucleated cell count = 9 cells/µL, 60% small lymphocytes and 40% large mononuclear cells; and total protein = 48 mg/dL, total nucleated cell count = 11 cells/µL, 67% small lymphocytes and 33% large mononuclear cells). Seven dogs had protein cytologic dissociation (total protein range: 33–174.1 mg/dL, total nucleated cell count range: 1–6 cells/µL). Two dogs had normal CSF, and one sample was non-diagnostic (sample clotted). No neoplastic cells were noted.

Twelve of thirteen dogs received some treatment, while one dog was euthanized on the day of diagnosis. Ten of twelve dogs were treated palliatively, including four dogs euthanized within one week of diagnosis, one dog with no available follow-up information, and one dog lost to follow-up 2 months after diagnosis. The remaining four palliatively treated dogs had a median survival time of 3.5 weeks ranging from 2 weeks to 2 months. One dog received palliative treatment along with palliative-intent radiation therapy and was euthanized 3 weeks after diagnosis because of neurologic deterioration to tetraplegia. One dog received palliative treatment, a C2–C3 modified dorsal laminectomy, and debulking surgery followed by definitive-intent radiation therapy and survived 3.5 months before being euthanized because of intractable pain.

### 3.3. NSTs External to the Spinal Canal

Nine of the thirty (30%) dogs were diagnosed with peripheral spinal NSTs, five being presumptive and four confirmed (Table 3). Seven tumors involved nerves or nerve roots of the brachial plexus, including five where the entire overtly apparent mass on MRI was distal to the intervertebral foramina and two where a portion of the mass was within one or more intervertebral foramen but with no spinal canal invasion. Two tumors involved the L7 nerve root, one being distal to the foramen and one with a component within the foramen.

The mean age was 8.3 ± 2.8 years, and the weight ranged from 7.0 to 37.0 kg, with a median of 31.5 kg. There were three spayed females, five neutered males, and one intact male. Breeds included mixed breed (n = 4), Labrador retriever (n = 2), Labradoodle (n = 1), pug (n = 1), and Havanese terrier (n = 1). The duration of clinical signs ranged from 1 week to 1 year. All dogs experienced progressive lameness of the affected limb. Physical and neurological examination abnormalities at presentation were limited to the affected limb and most commonly included monoparesis (n = 3), decreased reflexes (n = 6), muscle atrophy (n = 7), and lameness (n = 6).

Five dogs had EMG performed on the affected limb. All EMGs showed spontaneous activity consistent with denervation in multiple muscles of the affected limb; antebrachial musculature was more prominently affected compared with proximal limb muscles, and evaluation of the regional epaxial muscles was not reported. CSF analysis was performed in four dogs; two dogs had protein cytologic dissociation and two dogs had normal CSF. For the two abnormal samples, one sample was collected from the cisterna magna (total protein = 43 mg/dL, total nucleated cell count = 6 cells/µL), and one sample was collected via a lumbar puncture (total protein = 51.6 mg/dL, total nucleated cell count = 4 cells/µL).

All nine dogs received some form of treatment, though one dog was euthanized 3 days after diagnosis because of severe aspiration pneumonia presumed to be secondary to general anesthesia. Six dogs received only palliative treatment with a median survival time of 5.5 months ranging from 3 weeks to 14.5 months. Three of the six palliatively treated dogs had a portion of the mass that extended into the intervertebral foramen with survival times of 3 weeks, 1 month, and 4 months. Survival times in the three palliatively treated dogs without overt foraminal involvement were 7 months, 10 months, and 14.5 months. One dog received palliative treatment and radiation therapy and survived for 5 months. One dog received palliative treatment and limb amputation and survived 1 year. Neither dog that underwent radiation therapy or surgery had overt foraminal involvement on MRI.

## 4. Discussion

Nearly three-quarters of the dogs had NSTs involving one or more of the spinal nerves, and the majority of those arose from nerves or nerve roots associated with the brachial plexus. Prior studies of NSTs in dogs have rarely included both spinal nerve- and cranial nerve-associated NSTs together, making direct comparisons challenging. However, available data, including our results, underscore that the brachial plexus appears to be the anatomical region most commonly affected in dogs [5,8,9,15,24,29]. In people, NSTs typically occur in the head, neck, and extremities [4] with the majority also affecting the brachial plexus and the upper extremities [31]. The reason for the brachial plexus predilection has not been established in dogs. In people, previous trauma has been suggested as one explanation for why nerve sheath tumors have a propensity for certain locations [31]. NSTs have been reported to occur more commonly in the supraclavicular region of the brachial plexus [31], which has also been demonstrated to be more prone to injury compared with the nerves in the infraclavicular area [32]. The link between trauma and subsequent tumor development remains speculative. However, during wound healing in healthy individuals, it has been reported that there is an upregulation of genes typically associated with neurofibromatosis type 1 [31,33]. This is an autosomal dominant genetic condition in people that results in the formation of multiple small neurofibromas along peripheral nerves under the skin [34]. Neurofibromatosis type 1 is also associated with the development of malignant NSTs [31]. Thus, trauma or healing from trauma might mimic this predisposition in genetically unaffected individuals and could be explored in dogs.

Among the 13 invasive spinal cases in this series, there was a fairly even split between C1–C5 (7 cases) and C6–T2 (6 cases) locations. In contrast, all the peripheral spinal cases affected the C6–T2/brachial plexus region (seven cases) or the L7 nerve root (two cases). In another report on NSTs in dogs, just over half of the tumors in the brachial plexus region were peripherally located, while all thoracolumbar masses were noted to be invasive with an intradural location [9]. In reports focusing on spinal tumors that included NSTs (by definition, invasive spinal NSTs), the cranial to caudal location along the vertebral column was also variable with less of a clear predilection for the C6–T2/brachial plexus region [14,24,26,28]. While these studies varied in scope (any spinal tumor vs. NSTs, specifically), they are consistent with our findings and might suggest that NSTs located outside of the brachial plexus region are more likely to have canal invasion. This is likely attributed to the difficulty in identifying a peripherally located mass in the cranial cervical or thoracolumbar regions compared with those affecting nerves or nerve roots of the limbs, resulting in prominent lameness and visible limb muscle atrophy. Regardless, it is reasonable to include invasive spinal NSTs among the differential diagnoses for a characteristic mass located anywhere along the spinal canal.

While NSTs involving spinal nerves and nerve roots predominated, just over one-quarter of the dogs had tumors involving one or more cranial nerves, with only the trigeminal nerve being affected in the majority. This is consistent with multiple veterinary studies suggesting that the trigeminal nerve is the most common location for cranial NSTs [6,17,18]. The reason why the trigeminal nerve is preferentially affected in dogs is unknown, but possible contributing factors could be the large size and extensive branching of this nerve [35,36]. In people, trigeminal NSTs are rare [37,38], while acoustic neuromas, a type of NST affecting the vestibulocochlear nerve, are most common [39,40]. The reason for this discrepancy between dogs and people is not clear, but acoustic neuromas in people have been associated with prolonged exposure to loud noise [41]. Noise-induced inner ear trauma is likely less relevant for the majority of dogs outside of prolonged kennels or working situations [42,43]. Risk factors for cranial NSTs, especially trigeminal tumors, remain to be determined in dogs.

Antemortem definitive diagnosis of NSTs can be challenging. Magnetic resonance imaging characteristics used in the presumptive diagnosis of NSTs have been well described in dogs [15,17,18,19,20,23,24,25] with computed tomography or ultrasound occasionally utilized for diagnosis [10,15,21,22,44]. However, tumor biopsy samples typically used for definitive histologic diagnosis are uncommonly obtained unless animals are treated surgically or are submitted for necropsy. Seven dogs had cytology performed on the suspected nerve sheath tumors, one via squash preparation of an intra-operative sample and the other six via ultrasound-guided FNA. Cytology supported a diagnosis of malignant neoplasia in one dog and a sarcoma in three dogs, with an NST later confirmed histologically and immunohistochemically in one dog. The use of FNA and the cytologic features of NSTs have been minimally described in dogs [10,45]. In a prior small case series, cytology alone was not considered sufficient but rather aided in definitive diagnosis when combined with historical, clinical, and ultrasonographic features compatible with NSTs [45]. While only one of our cytology samples was confirmed histologically, our findings also support that FNA cytology can be useful adjunctive information in the diagnosis of NSTs. Additionally, access is not limited to peripherally located axillary masses as samples were also obtained from a mass affecting cranial nerves IX–XI (non-diagnostic), an invasive spinal mass affecting C7–T2 nerve roots (diagnostic for malignant neoplasia), and an L7 nerve root mass (diagnostic for a sarcoma). While cytologic analysis in a larger series of dogs with histologically confirmed NSTs would be necessary to determine the ultimate utility, it is reasonable to consider ultrasound-guided FNA as a minimally invasive tool that might aid in the diagnosis of NSTs where there is at least a peripherally accessible component to the mass.

The role of additional diagnostic tests in the diagnosis of NSTs is variable across studies and not clearly established. Electromyographic abnormalities in limb muscles have been reported in the vast majority of dogs with NSTs affecting thoracic or pelvic limb nerve roots [15,46]. Additionally, dogs with NSTs located within the intervertebral foramina or vertebral column had EMG changes in adjacent epaxial musculature, suggesting that EMG can be used to distinguish the proximal extent of suspected NSTs [15]. While EMG was abnormal in the affected limbs of the dogs in which it was performed, only five dogs in this case series had EMG, and findings for the epaxial musculature were not detailed, limiting any conclusions that can be drawn.

Cerebrospinal fluid analysis was abnormal in approximately three-quarters of the 18 dogs in which it was performed. However, across all tumor locations, findings were non-specific, characterized by protein elevation with or without a mild pleocytosis, and no samples contained neoplastic cells. Additionally, CSF results were normal in three dogs, including two with invasive spinal NSTs and one with a peripheral spinal NST with extension into the intervertebral foramina. Prior reports of CSF analyses in dogs with NSTs are limited but suggest similarly non-specific findings ranging from normal CSF to either protein cytologic dissociation or typically a mild pleocytosis [5,6,18]. While not performed in all dogs, CSF does not appear to be helpful in definitive diagnosis or in determining the extent of involvement in spinal NSTs and might be similarly limited in cranial NSTs. Therefore, the utility of CSF analysis might be primarily to help rule out other differentials such as an inflammatory process or types of neoplasia where cell exfoliation is more common.

Peripheral nerve sheath tumors generally carry a poor prognosis. Among dogs in this case series surviving a week after diagnosis, the median survival time was 4 months, though individual times varied from 2 weeks to more than 2 years and widely overlapped between cranial NSTs and all spinal NSTs. In prior case series of trigeminal NSTs or spinal NSTs in dogs receiving various therapies, survival times were also broad. These survival times ranged from days to years after diagnosis [5,6,14,17,18,25,26,29,47,48]. While the involvement of cranial nerve(s) versus spinal nerve(s)/nerve root(s) does not, by itself, predict tumor behavior or progression in dogs, the degree of NST invasion into the central nervous system might be important. Considering the palliatively treated dogs, survival times were generally shorter in dogs with masses with spinal canal invasion, foraminal involvement, or intracranial extension compared with dogs with masses without overt spinal canal, foraminal, or intracranial involvement, respectively. Peripherally located spinal NSTs have been previously reported to have longer survival times compared with dogs with more proximally situated tumors, though no significant differences were detected between dogs with masses affecting the plexus region and the nerve roots in the foramina [5]. Survival time for trigeminal NSTs was also not found to be significantly different between dogs with and without intracranial signs [17]. The impact of intracranial involvement was not possible to discern in other studies of trigeminal NSTs since most, if not all, dogs were reported as having masses with intracranial involvement [6,18,25,47]. As small numbers precluded statistical comparisons of subgroups in our study and many prior studies only focused on individual subgroups, further investigation is warranted regarding the influence of intracranial, foraminal, or spinal canal involvement on prognosis in dogs with NSTs.

The type of treatment pursued likely influences the outcomes of dogs with NSTs. While only a small number of dogs in this case series were treated with surgery and/or radiation therapy, half had survival times of a year or more. However, two dogs with invasive spinal NSTs managed aggressively only survived for 3 weeks and 3.5 months after diagnosis, and two palliatively treated dogs (one cranial NST and one peripheral spinal NST) each survived for a year or more. Among dogs with trigeminal NSTs treated with radiation therapy, survival times have been reported to be prolonged [6,25,47,49], but mean survival times were not significantly different in a series of dogs treated with radiation therapy compared to unirradiated dogs [17]. In dogs with spinal NSTs including some invading the canal, definitive treatments have also been suggested to prolong survival [14,29,48], especially if non-infiltrated margins are achieved for surgically managed cases [48]. However, direct comparisons to palliatively treated dogs are limited, and a subset of dogs with spinal NSTs still had poor outcomes despite aggressive therapy [5,26,28,29]. Surgery and/or radiation therapy might offer the best prognosis, but patient selection and other factors likely influence individual responses to treatment.

The limitations of this study mainly relate to the lack of definitive diagnosis in all cases and weaknesses associated with retrospective studies. Given the retrospective nature of this study, variability in treatments administered, and small sample size, we chose to present data descriptively. This decision avoided overstating any potential relationships but precluded investigation of certain aspects such as the impact of specific treatment protocols. While outcome information was simple (alive, dead, or euthanized), the lack of a standardized follow-up protocol might have resulted in recall bias. Selection bias likely also impacted individual outcomes such as the dog with the longest survival who underwent three rounds of radiation therapy, an option not feasible or pursued by most owners. Additionally, most dogs in this study were presumptively diagnosed via compatible clinical signs and imaging findings, with few confirmed by histopathology. It is possible that some of the tumors were other types of neoplasia with overlapping imaging characteristics such as lymphoma or meningioma or even non-neoplastic considerations such as neuritis. The lack of definitive diagnosis and the potential for misclassification of some dogs might therefore have impacted our interpretation of outcomes. This substantially limits the reliability and generalizability of our findings, and thus our results should be interpreted with caution. However, since antemortem diagnosis for NSTs commonly remains presumptive, the dogs in this case series still offer a clinically relevant population. Future larger multi-institutional studies could be designed to investigate predictors of outcome for dogs with confirmed NSTs.

## 5. Conclusions

Diagnosis of NST is typically suspected based on MRI, but FNA of the mass lesion is simple to perform and might aid in diagnosis. While conclusions are limited because most dogs had a presumptive diagnosis, the overall outcome for all NSTs was poor with a median survival of 4 months, and dogs were typically euthanized because of disease progression. Dogs with cranial NSTs or peripheral spinal NSTs typically survived longer than dogs with masses with spinal canal invasion, but our results highlight that various factors might impact the prognosis for dogs with NSTs including tumor location relative to the central nervous system and the type of treatment pursued.

## Figures and Tables

**Table 1:** Clinical and outcome information for dogs with cranial NSTs. * Dog 8 was alive at the time of last contact 18 months after diagnosis but was subsequently lost to follow-up. CN: cranial nerve, RT: radiation therapy.

Dog	Age (y)	Mass Location	Intracranial Clinical Signs	Intracranial Extension on MRI	Duration of Signs (wk)	Presumptive or Confirmed Diagnosis	Treatment	Definitive Treatment Details	Outcome	Survival Time (mo)
1	4	CN IX, X, XI	Yes	Yes	12	Confirmed	None		Euthanasia	0.07
2	12	CN IX, X, XI	Yes	Yes	4	Presumptive	Palliative		Euthanasia	4
3	6	Right CN V	No	No	40	Confirmed	Palliative		Euthanasia	23
4	10	Left CN V	No	No	3	Presumptive	Definitive	Definitive RT	Euthanasia	32
5	7	Left CN V	Yes	Yes	2	Presumptive	Palliative		Euthanasia	4
6	11	Right CN V	Yes	Yes	52	Presumptive	Palliative		Euthanasia	0.75
7	10	Right CN V	No	No	12	Presumptive	Palliative		Euthanasia	5
8	8	Left CN V	No	Yes	52	Presumptive	Definitive	Definitive RT	Lost to follow-up	>18 *

**Table 2:** Clinical and outcome information for dogs with invasive spinal NSTs. * Dog 10 had no contact after discharge following diagnosis. ** Dog 13 was alive at the time of last contact 2 months after diagnosis but was subsequently lost to follow-up. I: intradural; E: extradural; RT: radiation therapy.

Dog	Age (y)	Mass Location	Spinal Cord Clinical Signs	Mass Location within Canal on MRI	Duration of Signs (wk)	Presumptive or Confirmed Diagnosis	Treatment	Definitive Treatment Details	Outcome	Survival Time (mo)
1	12	Right C1–C2	Yes	I + E	1.4	Presumptive	Definitive	Definitive RT	Euthanasia	0.75
2	7	Right C1–C2	Yes	I + E	2	Presumptive	Palliative		Euthanasia	0.14
3	8	Right C2–C3	Yes	I + E	7	Confirmed	Definitive	Debulking surgery + RT	Euthanasia	3.5
4	9	Right C2–C3	Yes	I + E	3.5	Presumptive	None		Euthanasia	0
5	6	Left C3–C4	Yes	E	6	Presumptive	Palliative		Euthanasia	1
6	12	Left C3–C4		I + E	52	Presumptive	Palliative		Euthanasia	0.5
7	8	Right C4–C5	Yes	I + E	6	Presumptive	Palliative		Euthanasia	2
8	5	Left C6–C7	No	I + E	16	Presumptive	Palliative		Euthanasia	0.75
9	9	Left C6–C7	Yes	I	0.7	Presumptive	Palliative		Euthanasia	0.11
10	11	Left C7	Yes	I + E	8	Presumptive	Palliative		Lost to follow-up	0 *
11	11	Left T1–T3	Yes	I	12	Presumptive	Palliative		Euthanasia	0.11
12	6	Right C7–T2	Yes	I + E	16	Presumptive	Palliative		Euthanasia	0.18
13	11	Right T2–T3	No	I + E	4	Presumptive	Palliative		Lost to follow-up	>2 **

**Table 3:** Clinical and outcome information for dogs with peripheral spinal NSTs. RT: radiation therapy.

Dog	Age (y)	Mass Location	Foraminal Extension on MRI	Duration of Signs (wk)	Presumptive or Confirmed Diagnosis	Treatment	Definitive Treatment Details	Outcome	Survival Time (mo)
1	13	Right brachial plexus	No	36	Presumptive	Palliative		Euthanasia	14.5
2	8	Right brachial plexus	No	8	Presumptive	Palliative		Euthanasia	0.11
3	11	Left brachial plexus	No	8	Presumptive	Definitive	Definitive RT	Euthanasia	5
4	5	Right C7–T1 nerve roots	Yes	44	Confirmed	Palliative		Euthanasia	1
5	9	Left C8–T1 nerve roots	No	16	Presumptive	Palliative		Euthanasia	7
6	10	Right C8–T1 nerve roots	Yes	52	Presumptive	Palliative		Euthanasia	4
7	4	Left T1 nerve root	No	24	Confirmed	Definitive	Amputation	Euthanasia	52
8	8	Right L7 nerve root	Yes	8	Presumptive	Palliative		Euthanasia	0.75
9	8	Left L7 nerve root	No	1	Presumptive	Palliative		Euthanasia	10

## Data Availability

The data presented in this study will be made available by the authors on reasonable request.
